# The effects of sun exposure on colorant identification of permanently and semi-permanently dyed hair

**DOI:** 10.1038/s41598-023-29221-8

**Published:** 2023-02-07

**Authors:** Aidan Holman, Dmitry Kurouski

**Affiliations:** 1 Department of Entomology, Texas A&M University, College Station, TX 77843 USA; 2 Department of Biochemistry and Biophysics, Texas A&M University, College Station, TX 77843 USA; 3 Department of Biomedical Engineering, Texas A&M University, College Station, TX 77843 USA; 4 Institute for Advancing Health through Agriculture, College Station, TX 77843 USA

**Keywords:** Applied optics, Optical sensors, Environmental chemistry

## Abstract

During bloating and active decay, human remains begin to deform and warp their physical identity. After the skin and muscles loosen and detach from their skeletal structuration, everything but bones, teeth, and hair will fully disintegrate into the soil that surrounds the body. Nearly half of people in the world dye their hair with a variety of permanent and semi-permanent colorants. Expanding upon this, we hypothesized that confirmatory analysis of hair colorants can be used to facilitate and advance forensic analysis of human remains. A growing body of evidence suggests that hair colorants can be identified directly on hair using surface-enhanced Raman spectroscopy (SERS). In this study, we investigate the extent to which SERS can be used to detect black and blue permanent and semi-permanent dyes on hair exposed to sunlight. Our results showed that although substantial photodegradation of all dyes was observed by week 7, SERS enabled highly accurate detection and identification of hair colorants during all 10 weeks of hair exposure to the sunlight with on average 99.2% accuracy. We also found that SERS could be used to predict fading rates of hair colorants. This information can shed light on the exposure of human remains to the exterior environment.

## Introduction

The sun is an impetus for chemical and physical changes of human remains. Within a few hours after death, the human body starts exhibiting bloating followed by active decay and ultimately a complete decomposition of soft tissues. At the same time, bones, teeth, and hair can remain intact for years^1^. Anthropological examination of teeth and bones can be used to determine the age and sex of a decedent, identify post-mortem intervals and even predict possible causes of death^1,2^. Although hair can provide additional details about the decedent, is often overlooked within the forensic analysis of human remains. Currently-used microscopic analysis of hair is highly subjective and inconclusive^3,4^. In most cases, microscopic examination of hair can be used to identify a body area from where the hair came from and the race of the individual^5,6^. These limitations catalyzed a search for more advanced analytical tools that can empower forensic analysis of hair^7,8^.

Hair is often colored using permanent or semi-permanent dyes^8^. The former type of colorants requires developers that oxidize aromatic diamines into aromatic polymers, known as Bandrowski’s bases^9,10^. Upon oxidation, these molecules interact with keratin diffusing deep into the hair cortex, which makes them last on hair for months. Semi-permanent dyes are aromatic molecules with a large number of conjugated double bonds. These colorants do not require developers. Therefore, semi-permanent dyes easily come off upon hair washing^11^. Confirmatory analysis of hair colorants can be used to establish a connection between a decedent and a crime scene, as well as help to identify the decedent’s identity.

A growing body of evidence shows that surface-enhanced Raman spectroscopy (SERS) can be used to detect and identify colorants present on hair^8,12,13^. In SERS, noble metal nanostructures are used to enhance Raman scattering of molecules located in proximity to metallic surfaces^14^. Surface enhancement can reach 10^6^–10^8^, enabling single-molecule sensitivity of SERS^15,16^. Consequently, if gold nanoparticles are applied to hair and illuminated by light, vibrational fingerprints of colorants present on hair, and their chemical structure, can be determined within seconds^17,18^. In 2015, Kurouski and Van Duyne demonstrated that SERS could be used to differentiate between permanent and semi-permanent colorants present on hair^8^. Esparza and co-workers showed that SERS was able to identify underlying colorants if the hair was re-dyed afterward^13^. Recently, Higgins and Kurouski demonstrated that SERS enabled confirmatory identification of more than 30 different colorants^12^. It was also shown that SERS could be used to predict the color and brand of colorants present on hair.

The question to ask is whether environmental factors, such as sun exposure, can damage dyes present on hair and, consequently, lower the accuracy of SERS-based identification of permanent and semipermanent colorants. Light-driven degradation or fading of colorants is an active area of research in art conservation science^19^. Using SERS, Pozzi and co-workers were able to determine pigment compositions used by Van Gogh^20^. In 1888, the artist began to craft the first of three paintings known as *Bedroom*. In a letter he wrote to his brother, he described the painting as “walls pale lilac, the floor in a broken and faded red…”. However, nowadays the walls of the *Bedroom* are not lilac but blue, concurrently–the floor is not red but appears more cyan and light purple. These drastic changes in the colorants of *Bedroom* are caused by their photodegradation. Pozzi and co-workers discovered that Van Gogh used cochineal red mixed with a blueish pigment for the walls. Light cased fading of cochineal red; with no red left to combine with blue to make a purple (or “lilac”), all that remained was the blue we see today^20^.

In the current study, we examine the extent to which sunlight can lower the accuracy of SERS-based detection of both permanent and semi-permanent, black and blue dyes on strands of hair. We also investigate whether SERS can be used to predict the time of hair exposure to the sun, which, in turn, can *shed light* on the exposure of human remains to the external environment.

## Results and discussion

**Figure 1:**
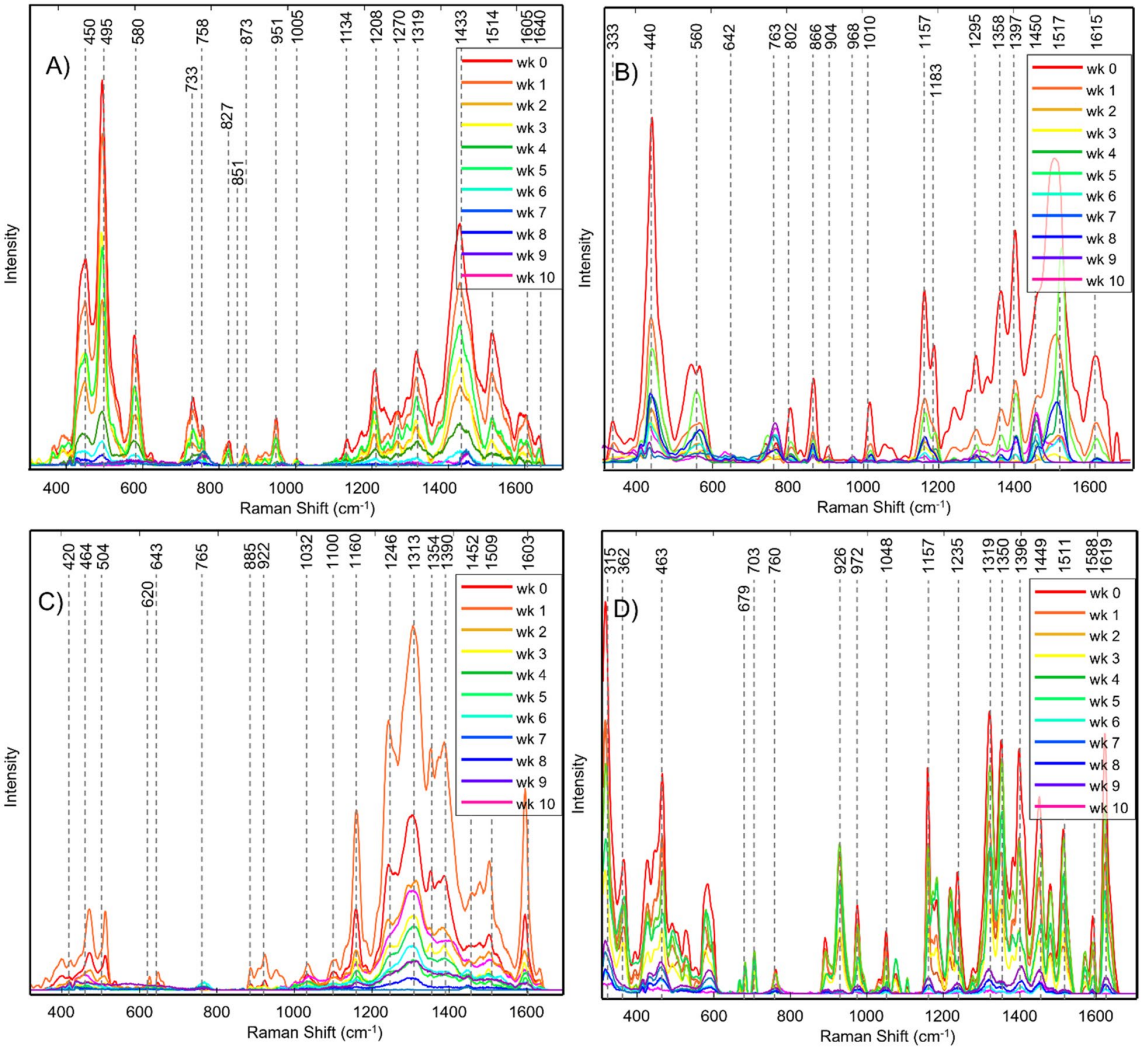
Averaged SERS spectra of (**A**) BLK^P^, (**B**) BLU^P^, (**C**) BLK^S^, and (**D**) BLU^S^ at week 0 to week 10 of hair exposure to sunlight.

In the SERS spectra acquired from BLK^P^ hair at week 0, we detected vibrational bands centered at 450, 495, 580, 733, 758, 827, 851, 873, 951, 1005, 1134, 1208, 1270, 1319, 1433, 1514, 1605 and 1640 cm^–1^, Figs. 1 and S1. We found that as time progressed, the intensity of these vibrations decreased. At week 7, the intensities of all vibrational bands were comparable to the noise level in the acquired SERS spectra. In the spectra acquired from BLU^P^ hair at week 0, we observed vibrational bands centered at 330, 440, 560, 642, 763, 802, 866, 904, 868, 1010, 1157, 1183, 1295, 1358, 1397, 1450, 1517, and 1615 cm^–1^, Figs. 1 and S1. Similar to BLK^P^, we observed a drastic decrease in the intensity of these bands at week 6 of hair exposure to sunlight. Nevertheless, all bands remained detectable in the SERS spectra of BLU^P^ hair at weeks 6–9. Finally, only one band at 1449 cm^-1^ was detectable in the SERS spectrum of BLU^P^ hair at week 10. These spectroscopic changes show progressive photodegradation of both BLK^P^ and BLU^P^ under sunlight.

In the SERS spectra acquired from BLK^S^ hair at week 0, we observed vibrational bands at 450, 495, 580, 733, 758, 827, 851, 873, 951, 1005, 1134, 1208, 1270, 1319, 1433, 1514, 1605 and 1640 cm^−1^, Figs. 1 and S1. Similar to other colorants, intensities of these bands decreased as the time of the sun exposure of the colored hair progressed. At week 6, we observed only three (495, 1313, and 1452 cm^−1^) out of all discussed above vibrational bands. It should be noted that in the SERS spectra collected from BLU^S^, we observed a change in the relative intensities of vibrational bands at 733 and 758 cm^−1^. As time progressed, intensity of 733 cm^−1^ decreased, whereas the intensity of 758 cm^−1^ increased. These spectroscopic changes point to the light-induced changes in the chemical structure of BLU^S^.

In the SERS spectrum acquired from BLU^S^ hair at week 0, we observed vibrational bands at 333, 440, 560, 642, 763, 802, 866, 904, 968, 1010, 1157, 1295, 1358, 1397, 1450, 1517, and 1615 cm^−1^, Figs. 1 and S1. Intensities of these bands decreased as the time progressed, however, most of these bands were detectable in the SERS spectra acquired at weeks 8–10. These findings showed that BLU^S^ is substantially more resistant to photodegradation compared to other colorants used in our work. These results also suggest that machine learning rather than visual analysis of complex changes in the vibrational bands of colorants should be used to identify BLK^P^, BLU^P^, BLK^S^, and BLU^S^ on hair at different exposures of hair to sunlight.

Therefore, we utilized PLS-DA to determine the accuracy of SERS-based identification of colorants on hair exposed to sunlight for up to 10 weeks, Table 1. We found that all colorants could be highly accurately (95.5–100%) identified on hair exposed to sunlight at any week. Specifically, our PLS-DA model enabled 100% identification of all colorants at week 0, 3, 4, 6, 7 and 10, whereas 97–99% accuracy hair colorants could be detected at weeks 1, 2, 5, and 8. Finally, at weeks 5 and 9, analyzed hair colorants could be correctly predicted with 99% and 99.5% accuracies, respectively. These results demonstrate that SERS can be used to predict colorants on hair exposed to the sunlight for up to 10 weeks, as well as predict the duration of sun exposure on hair.

**Table 1:** Class prediction models of each dye for each week, featuring the true positive rate (TPR) indicating the accuracy of the program to predict that color among the rest and the overall success rate (OSR) of the program’s ability to predict all colors each week.

		Color/dye			
		BLK^P^	BLU^P^	BLK^S^	BLU^S^
Sampling period	OSR (%)	TPR (%)			
Week 0	100	100	100	100	100
Week 1	98	100	94	98	100
Week 2	97.5	100	100	90	100
Week 3	100	100	100	100	100
Week 4	100	100	100	100	100
Week 5	99	98	100	100	98
Week 6	100	100	100	100	100
Week 7	100	100	100	100	100
Week 8	97.5	98	100	92	100
Week 9	99.5	100	98	100	100
Week 10	100	100	100	100	100
*Overall*	99.2	99.6	99.3	98.2	99.8

**Figure 2:**
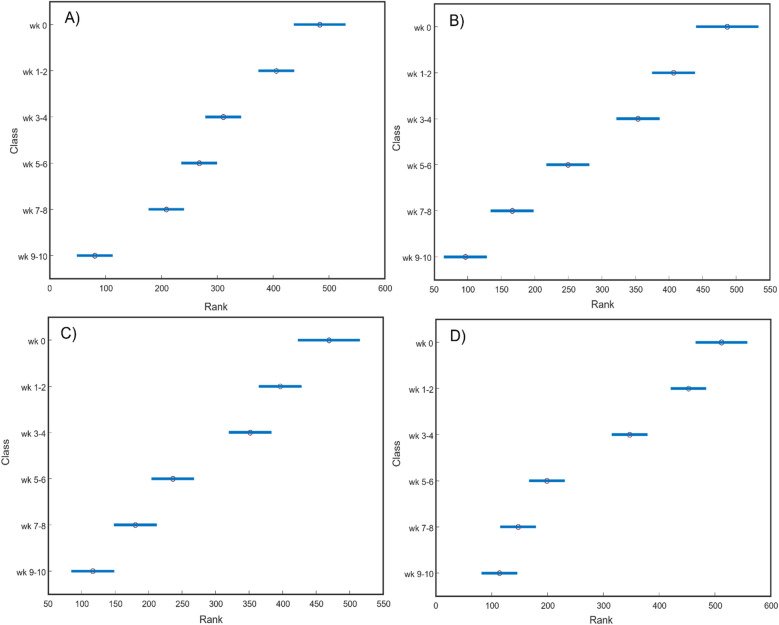
ANOVA graphs for (**A**) BLK^P^ at 1433 cm^–1^, (**B**) BLU^P^ at 1157 cm^–1^, (**C**) BLK^S^ at 921 cm^–1^ and (**D**) BLU^S^ at 1157 cm^–1^.

The question to ask is if any of the discussed above vibrational bands could be used to monitor dye fading. ANOVA results show that 1433 cm^–1^ band can be used to monitor photodegradation of BLK^P^, whereas the 1157 cm^–1^ band could be used to determine the duration of sun exposure on BLU^P^, BLU^S^, respectively. Finally, we found that 921 cm^–1^ band can be used to track fading of BLK^S^, Fig. 2. We also found that other vibrational bands in the SERS spectra of BLK^P^, BLU^P^, BLK^S^, BLU^S^ colorants show similar graduate decrease in the intensity, Fig. S2. These results demonstrate that SERS could be used to monitor fading of the colorants on hair. This information can be used to predict the duration of the exposure of human remains to the exterior environment.

## Conclusions

We found that sunlight caused fading of both blue and black permanent and semi-permanent colorants on hair. SERS analysis of hair showed that most of these dyes were substantially photodegraded by week 7. Nevertheless, utilization of PLS-DA allowed for highly accurate identification of hair colorants with on average of 99.2% accuracy of any week of hair exposure to the sunlight. PLS-DA also enables highly accurate identification of the duration of colorant exposure to sunlight. Furthermore, our results show that the intensity of vibrational bands of the colorants could be used to track photodegradation of colorants. Future research that analyzes and compares a broader range of colors and types of dyes, such as temporary and demi-permanent, is required to further understand the effect of sunlight on SERS-based analysis of colorants. Additionally, it is important to investigate the effects of other environmental conditions that hair can be exposed to such as chlorine in pool water, ecosystems found in natural water bodies, and dangerous elements found in local water utilities. These studies will improve the accuracy of SERS in forensic hair analysis.

## Materials and methods

### Experiment

#### Hair dyes

Ion Jet Black (No. 305730), Ion Black (No. 305052), Ion Tanzanite (No. 405607), and Ion Sapphire (No. 405068) were purchased from a local Sally Beauty supply store (College Station, TX). Each colorant was applied on blonde, Caucasian, virgin hair according to instructions provided on the colorant package box. In total, we had four groups of samples: (1) black permanent (BLK^P^), (2) blue permanent (BLU^P^), (3) black semi-permanent (BLK^S^), and (4) blue semi-permanent (BLU^S^).

Colored hair was placed outside a west-facing window. Samples were collected every week at ~ 8 pm by cutting off 1–1.5 inches of hair. After cut, each sample was placed in a clean, dry plastic tube. Maximum UV indices were recorded daily using the EPA’s SunWise UV Index app endorsed by the United States Environmental Protection Agency (USEPA) and the National Oceanic and Atmospheric Administration (NOAA). Daily high and average temperatures, as well as the amount of daytime, were also recorded using data by NOAA (NOAA 2021), Fig. S3.

### Raman spectroscopy

Prior to spectral acquisition, 5 µL of a suspension of gold nanoparticles (AuNPs) was applied to hair. Surface-enhanced Raman spectra were collected from 1–2 hair strands using a home-built confocal inverted microscope (Nikon, Model TE-2000U). Laser light was generated using a 785 nm solid-state laser (Necsel SLM785.0-FS-01) and focused on the sample using a 20 × Nikon objective. Scattered light was collected using the same objective and then directed to Princeton Instruments spectrograph equipped with a 600-groove/mm grating. Prior to entering the spectrograph, elastically scattered photons were cut off using a long-pass filter. Inelastically scattered photons were collected using a thermoelectrically cooled PIX-400BR CCD camera (Princeton Instruments). Laser power at each sample was ~ 1.8 mW. Spectral action time varied between 10 and 18 s.

### Data analysis

Spectral processing and averaging were conducted using PLS_Toolbox 8.6.2 (Eigenvector Research, Inc., Manson, WA). Raman spectra were imported into Matlab (Mathworks), area-normalized, and baseline-corrected for preprocessing. For PLS Discriminant Analysis (PLS-DA), “suggested” models were utilized and displayed as follows.

## Supplementary Information

Supplementary Figures.

## Data Availability

The datasets used and/or analyzed during the current study available from the corresponding author on reasonable request.
