# Supplementary material for: The effects of sun exposure on colorant identification of permanently and semi-permanently dyed hair
Source: Sci Rep. 2023 Feb 7;13:2168. doi: 10.1038/s41598-023-29221-8 (PMC9905578; doi:10.1038/s41598-023-29221-8)
Supplement: Supplementary file 1 — Supplementary Figures. [file 41598_2023_29221_MOESM1_ESM.docx]

The Effects of Sun Exposure on Colorant Identification of Permanent and Semi-Permanently Dyed Hair

Aidan Holman^1,2^ and Dmitry Kurouski^2,3,4*^

*E-mail: dkurouski@tamu.edu Tel: 979-458-3778.

ORCID

Dmitry Kurouski: 0000-0002-6040-4213

Aidan Holman: 0000-0003-4244-7348

1. Department of Entomology, Texas A&M University, College Station, Texas 77843, United States
2. Department of Biochemistry and Biophysics, Texas A&M University, College Station, Texas 77843, United States
3. Department of Biomedical Engineering, Texas A&M University, College Station, Texas, 77843, United States
4. Institute for Advancing Health through Agriculture, College Station, Texas, 77843, United States

**Supporting Information**

(A)

(B)

(C)

(D)****

**Figure S1.** Averaged SERS spectra (solid lines) with corresponding standard deviations (colored areas) acquired from BLK^P^ (A), BLU^P^ (B), BLK^S^ (C) and BLU^S^ (D) before sun exposure (week 0, red) and after 3 (green), 6 (blue), and 10 (yellow) weeks.

**Figure S2.** ANOVA of vibrational bands in the acquired SERS spectra of P-Black, P-Blue, S-Black and S-Blue before (wk 0) and after sun exposure for 1-2, 3-4, 5-6, 7-8, and 9-10 wks.

**
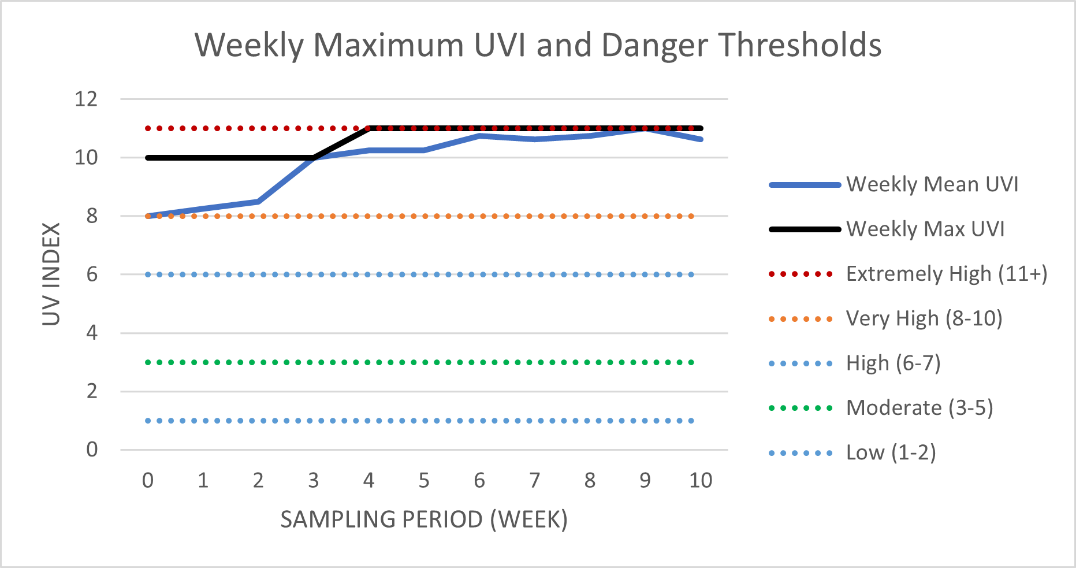

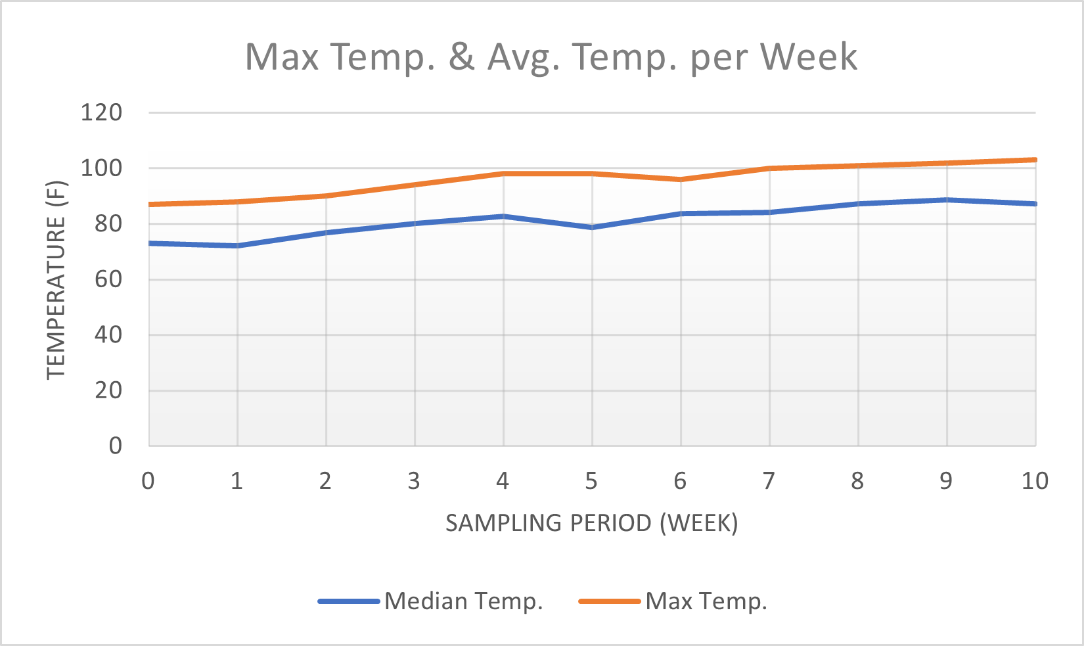
**

**Figure S3. (**Top) Largest (blue) and mean (black) maximum UVI per week. (Bottom) Median temperature (blue) and maximum temperature (orange) per week
